# Attitude of Parents of Children with Cerebral Palsy Towards COVID-19 Vaccination

**DOI:** 10.3390/ijerph20031909

**Published:** 2023-01-20

**Authors:** Ramy Mohamed Ghazy, Malik Sallam, Noha Fadl, Etwal Bouraad, Naglaa Youssef, Omnya Samy A. Ghoneim

**Affiliations:** 1 Tropical Health Department, High Institute of Public Health, Alexandria University, Alexandria 21561, Egypt; 2 Department of Pathology, Microbiology and Forensic Medicine, School of Medicine, The University of Jordan, Amman 11942, Jordan; 3 Department of Clinical Laboratories and Forensic Medicine, Jordan University Hospital, Amman 11942, Jordan; 4 Family Health Department, High Institute of Public Health, Alexandria University, Alexandria 21561, Egypt; 5 Department of Epidemiology and Population Health, American University of Beirut, Beirut 961, Lebanon; 6 School of Pharmacy, Lebanese International University, Beirut 961, Lebanon; 7 Department of Medical-Surgical Nursing, College of Nursing, Princess Nourah Bint Abdulrahman University, Riyadh 11671, Saudi Arabia; 8 Department of Physical Therapy for Pediatrics and Pediatric Surgery, Faculty of Physical Therapy, Badr University in Cairo, Cairo 11829, Egypt

**Keywords:** cerebral palsy, COVID-19 vaccine, vaccine hesitancy, children, parental attitude, Egypt, SARS-CoV-2

## Abstract

Children with cerebral palsy (CP) are at a greater risk of respiratory complications from coronavirus disease 2019 (COVID-19). Therefore, this study aimed to assess COVID-19 vaccine hesitancy (VH) among parents of CP children in Egypt, using the Arabic version of the Parental Attitude about Childhood Vaccination (PACV) questionnaire. This cross-sectional survey study was conducted at the outpatient clinics of two hospitals in Cairo, Egypt. Parents of children with CP were recruited using a simple random sampling technique. A total of 321 parents were enrolled; more than half of them were mothers of the children (61.37%); and the majority were Egyptians (87.23%) and living in urban areas (84.42%). Nearly 70% of the parents were hesitant to administer the COVID-19 vaccine to their children. A multiple linear regression model revealed that the PACV mean scores were lower among the following categories: (1) parents who could pay back loans, compared to those who could not pay back loans and who reported insufficient income (β = −2.39, *p* = 0.030); (2) non-Egyptian parents (β = −1.54, *p* = 0.002); (3) those who were fully vaccinated against COVID-19 themselves or had the intention to receive the complete COVID-19 vaccination (β = −6.28, *p* < 0.001); (4) those who had the intention to give the COVID-19 vaccination to their children (β = −3.04, *p* < 0.001); and (5) parents whose children received routine vaccines (β = −2.86, *p* < 0.045). After adjusting for other covariates, the parental COVID-19 vaccine status (β = −6.28, *p* < 0.001) and parents who experienced a COVID-19-related death in the family (β = −1.75, *p* < 0.001) showed significantly lower mean PACV scores. However, higher mean PACV scores were reported among parents who had a COVID-19 infection (β = 2.77, *p* < 0.001) or who were not sure (β = 2.94, *p* < 0.001). Our findings suggest the need to increase COVID-19 vaccine acceptance among parents of vulnerable children to reduce the negative consequences of COVID-19.

## 1. Introduction

The severe acute respiratory syndrome coronavirus 2 (SARS-CoV-2) is a virus that causes the potentially fatal coronavirus diseases 2019 (COVID-19), which mostly affects the respiratory system [1], with recognizable yet variable patterns of morbidity and mortality [2,3]. Moreover, the COVID-19 pandemic affected all aspects of human life [4,5,6] and caused catastrophic economic recession [7]. The World Health Organization (WHO) declared COVID-19 a global pandemic on 11 March 2020 [8]. On 4 January 2023, approximately 655.7 million cumulative cases, with 6.7 million deaths due to COVID-19, were reported globally. In Egypt, 515,533 cases of COVID-19 and 24,802 deaths have been reported so far [9]. Other than vaccination, Egypt adopted different public health and social measures to contain the pandemic [10]. Since 24 January 2021, Egypt has distributed 101 million doses of COVID-19 vaccines, equaling nearly 98.7 total doses administered per 100 population [9]. The Egyptian Ministry of Health and Population began sending mobile teams to areas with low vaccination rates, supported by healthcare workers, to perform house visits in order to expand access to vaccines in rural settings and urge more individuals to get themselves and their children with ages >12 years vaccinated under family consent [11].

The Global Burden of Diseases estimates that over 53 million children have developmental disabilities (DDs), such as epilepsy, intellectual disability, cerebral palsy (CP), impairment of vision and hearing, autism, hyperactivity, and attention deficit. Nearly 95% of children with DDs live in low- and middle-income countries (LMICs) [12]. CP represents a group of heterogeneous and non-progressive neurodevelopmental disorders caused by disturbances in the developing brain [13]. The global prevalence of children with CP has been estimated at 1.2% or 12 per 1000 live births [14]. In Egypt, the estimate is higher at a rate of 2.04 per 1000 live births [15].

The disproportionate effect of SARS-CoV-2 infection on children with DDs might be related to the direct effect of the virus itself or the impact of the pandemic hindering access to essential health services [16]. Additionally, children affected by CP are at an increased risk of developing COVID-19 respiratory complications [17]. Previous research using data from 43,465 children aged ≤18 years old demonstrated that children with neurodevelopmental abnormalities were more likely than healthy ones to be hospitalized as a result of COVID-19 [18]. Thus, the Centers for Disease Control and Prevention (CDC) recommended COVID-19 vaccines for everyone aged six months and older, including those with DDs [19]. Vaccination against COVID-19 has been accepted as a crucial measure for controlling and limiting the risk of SARS-CoV-2 infection, including the emerging variants of the virus, due to its safety, immunogenicity [20], and efficacy [20]. However, parental vaccine hesitancy (VH) has been identified as an important obstacle to childhood vaccination [21]. VH is listed among the top 10 threats to global health [22]. VH is defined as a ‘’delay in the acceptance or refusal of vaccines despite their availability’’ [23]. Many parents are concerned about the COVID-19 vaccine’s safety and its potential harm, and this may increase their VH [24]. False and misleading claims about COVID-19 can negatively influence individuals’ attitudes towards vaccine uptake [25,26]. The ‘Parent Attitudes about Childhood Vaccines’ (PACV) is a well-established questionnaire that can be utilized efficiently to investigate parental COVID-19 VH. The PACV survey has been proven to be effective at identifying parents who are anti-vaccine and predicting future VH [27].

The COVID-19 pandemic’s impact on the health of children with CP is not well understood [28]. In addition, limited data are available on parental attitudes towards COVID-19 vaccines for children with CP in LMICs. Drawing attention to parents’ intention to vaccinate their children and the factors contributing to their intention is essential for enhancing policymakers’ and healthcare providers’ insights about the magnitude of the problem and its contributing factors. Hence, this study aimed to assess parental VH towards COVID-19 vaccination among their children with CP in Egypt using the PACV tool.

## 2. Materials and Methods

### 2.1. Study Design and Settings

A cross-sectional study was carried out over a two-month period (September to October 2022) at the outpatient physical therapy clinic of the Faculty of Physical Therapy, Badr University in Cairo, and Prof. Dr. Kamal Shoukry’s Pediatric Rehabilitation Center in Cairo, Egypt.

### 2.2. Target Population and Eligibility for Participation

Parents of children aged 5–12 years and diagnosed with CP for at least six months were included in the study. All children with terminal diseases or severe advanced comorbid conditions (i.e., cancer) were not eligible to participate. Parents with mental or communication disabilities were excluded from this study as well.

### 2.3. Sample Size and Sampling Technique

Based on a prevalence rate of 25% for the acceptance rate of COVID-19 vaccination among patients with a physical disability [29], using a 5% accepted degree of precision, an α of 0.05, a power of 95%, and a design effect of “1”, the minimum required sample size was 292 parents. The total sample size was increased by approximately 10% to compensate for the missing data and non-response. The required sample size was determined using the EPI-Info 7.2 software. A simple random sampling procedure was performed to obtain the required sample.

### 2.4. Questionnaire of Data Collection

A predesigned questionnaire was administered to gather data through face-to-face interviews with parents/caregivers (Supplementary File). The questionnaire consisted of the following three sections: Section I: Parents’ data included residence area, family size, age, education, occupation, and income. Moreover, questions related to COVID-19 were collected (i.e., parents’ history of COVID-19 infection, experienced COVID-related death among the family members, parental uptake of the COVID-19 vaccine, and perceived seriousness and susceptibility to the disease). Parents were also asked about the vaccination status of their children (including routine vaccination and influenza vaccine uptake) and their intention to vaccinate their children against COVID-19. Section II: Children’s data included age, sex, birth order, and chronic comorbidities. Section III: Parent Attitudes about Childhood Vaccines (PACV): The 15-item Arabic version of PACV was used to identify parental vaccine hesitancy [30]. PACV is a validated scale with three subdimensions: (i) behavior towards the vaccine, (ii) beliefs about the safety and efficacy of the vaccine, and (iii) attitudes and trust. Answers about scale questions were taken from three different response formats according to the types of items/questions: (1) closed-ended questions using a ‘Yes/No/I do not know’ response scale, (2) 5-item Likert scale items using ‘Strongly Agree/Agree/Not Sure/Disagree/Strongly Disagree’, and (3) an 11-point scale (0 to 10) of questions [27].

In order to calculate the total PACV score, items were recategorized as follows: (1) Responses to items with a 5-point Likert-scale were collapsed; (i) ‘strongly agree/agree’ were considered ‘non-hesitant’, and (ii) ‘strongly disagree/disagree’ were considered ‘hesitant.’ (2) Responses to items with a 5-point Likert-scale ranging from ‘not at all concerned’ to ‘very concerned’ were collapsed as well, and responses of ‘somewhat or very concerned’ were considered ‘hesitant’ responses, while responses of ‘not at all or not too concerned’ were considered ‘non-hesitant’ responses. (3) For items with a yes/no response, ‘yes’ was considered to be the ‘hesitant’ response and ‘no’ to be the ‘non-hesitant’ response. Only for one item, “If you had another infant today, would you want him/her to get all the recommended shots?’’ was the reverse true. (4) For the items with a 10-point Likert-scale, we considered 0–5 to be ‘hesitant’, 6–7 to be ‘not sure’, and 8–10 to be ‘non-hesitant’ responses. Responses were assigned a score of “2” for ‘*hesitant*’ responses, “1” for ‘*not sure*’ responses, and “0” for ‘*non-hesitant*’ responses. Unweighted item scores were summed to calculate a raw score, and a simple linear transformation was used to convert the raw score to a 0–100-point scale [27]. The mean PACV score was calculated, where a higher mean score indicated high parental hesitancy towards the vaccine. A pilot test was conducted to ensure the clarity and feasibility of the questionnaire.

### 2.5. Case Classification

Cerebral palsy is classified into spastic, dyskinetic, and ataxic subtypes. The spastic subtype is further divided into the hemiplegic and bilateral types. The bilateral type is subdivided into quadriplegia and diplegia [31].

Based on sitting, walking, and wheeled mobility, the Gross Motor Function Classification System (GMFCS), which uses a 5-level classification system, was utilized to assess the gross motor function of children with CP. Functional capacities, the necessity for assistive technology, such as portable mobility aids (walkers, crutches, or canes) or wheeled mobility, and, to a much lesser extent, the quality of movement, were used to differentiate between levels [31]. The researcher (OG) looked over the child’s medical records to determine the type of CP and the severity of GMFCS. [32]. The child’s medical records were reviewed by the researchers (R.M.G., OG) to obtain the type of CP and the level of GMFCS.

### 2.6. Statistical Analysis

The STATA package (version 16) was used to manipulate, visualize, and perform statistical analyses. The main outcome of the present study was parental VH towards vaccinating their children with the COVID-19 vaccine. Descriptive statistics using mean ± standard deviation (SD) and frequency distribution using percentage (%) were used to describe the sociodemographic data of parents and their children with CP. An independent t-test was performed to compare the mean PACV scores between the two unrelated groups. One-way ANOVA was used to compare the mean PACV scores between three or more unrelated groups. Odds ratios (OR) with 95% confidence intervals (CI) were reported. Independent variables with a *p* value ≤0.20 in the bivariate analysis were maintained and entered into a multivariate analysis that was conducted to verify their correlation with the dependent factor (PACV score). Additionally, the potential presence of multicollinearity was examined and ruled out. The findings were considered significant at a *p* value less than 0.05, with 95% CI.

### 2.7. Ethical Approval and Participants’ Rights

Ethical approval (NO: P.T.REC/012/003980) was obtained before data collection from the Ethics Committee of the Faculty of Physical Therapy at Cairo University. The Declaration of Helsinki was followed to maintain the rights of the parent/caregivers and their children. The parents/caregivers provided written informed consent after receiving clear information about the study and understanding its purpose. They were also informed that they could withdraw their consent because their participation was voluntary and that it would not have any adverse consequences on their children’s health or provided services. The confidentiality of the parents’ and their children’s data was protected by coding the collected data, and only the researcher could access the saved data.

## 3. Results

### 3.1. Characteristics of the Parents

Approximately two-thirds of the parents were the children’s mothers (61%), the majority were Egyptians (87.23%), and the majority were living in urban areas (84.42%). The studied parents were middle-aged, between 30–39 years (30.84%) and 40–49 years (31.46%) (Table 1).

### 3.2. Characteristics of Children with CP

A total of 321 children were recruited in the present study, with a mean age of 8.2 ± 2.11 years. Nearly half of them were females (55%), and around 40% were first- or second-order children. Approximately 40% of the children were diagnosed with diplegia, 24.9% were classified as GMFCS stage II, and 38.82% had a history of chronic diseases. A history of COVID-19 infection was reported in 14% of the children. Most of the participating children received routine vaccines (95.52%), whereas 43.4% received the influenza vaccine, as reported by their parents (Table 2).

### 3.3. Parental Beliefs and Attitudes Towards COVID-19 and Vaccination

**Figure 1:**
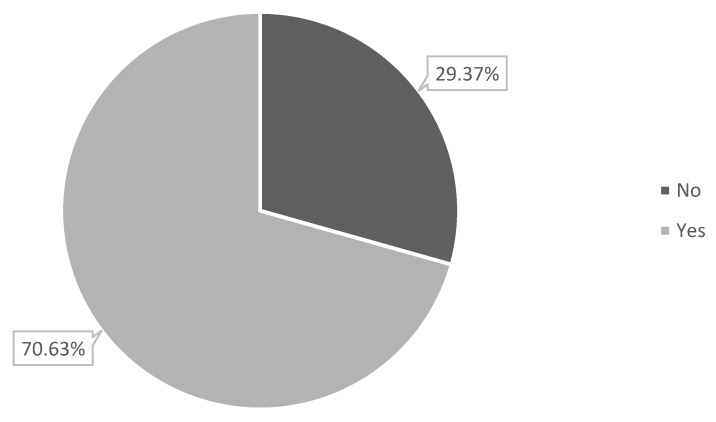
Parental hesitancy towards COVID-19 vaccination among children with CP.

Nearly half of the parents reported that they had been infected with COVID-19 (49%), etc., and 40.81% reported a history of COVID-19-related death in the family. Regarding parental COVID-19 vaccine status, 64.8% reported that they did not want to take the vaccine or to continue taking the booster doses, 91% strongly disagreed that COVID-19 was dangerous, and around 53% considered their children to be at high risk of contracting COVID-19 (Table 3). Based on the PACV, 70.63% of parents reported their hesitancy to give their children the COVID-19 vaccine (Figure 1).

### 3.4. Factors Correlated with Parental Hesitancy Towards COVID-19 Vaccination

The bivariate association between the mean PACV score and statistically significant covariates is presented in Table 4. Egyptian parents, those who had a history of COVID-19-related deaths in their families, incomplete or lack of parental COVID-19 vaccination, incomplete childhood routine immunization, and lack of parental intention to give their child the COVID-19 vaccine were significantly associated with higher mean PACV scores (*p* < 0.05) (Table 4).

### 3.5. Predictors of Parental Hesitancy Towards COVID-19 Vaccination

Table 5 presents the multiple linear regression analysis adjusted for child age, child order, work sector, childhood routine vaccine, and influenza vaccine. The mean score of PACV significantly decreased in parents who could pay back loans, compared to those who could not pay back loans and those who did not have enough income (β = −2.39, *p* = 0.03), non-Egyptians (β = −1.54, *p* = 0.002), and parents who were fully vaccinated or waiting to complete the COVID-19 vaccination, compared to those who did not want to have or complete it (β = −6.28, *p* < 0.001). Moreover, the mean score of PACV remarkably decreased in parents with an intention to give the COVID-19 vaccine to their children, compared to the counterpart group without the intention to give the vaccine to their children (β = −3.04,* p* < 0.001); the mean score also decreased in parents whose children received routine vaccines, compared to those whose children did not receive them (β = −2.86, * p* < 0.001). After adjusting for other covariates, the parental COVID-19 vaccine status (β = −6.28, *p* < 0.001) and parents who experienced a COVID-19-related death in the family (β = −1.75, *p* < 0.001) showed significantly lower PACV scores. However, higher PACV scores were reported among parents who had COVID-19 before (β = 2.77, *p* < 0.001) or who were not sure if they had COVID-19 (β = 2.94, *p <* 0.001), compared to parents who did not have COVID-19. In those who agreed that COVID-19 was a serious illness, the PACV score decreased, but the results were not significant (β = −1.04, *p* = 0.11).

## 4. Discussion

There are insufficient data in public health surveillance systems regarding the effect of COVID-19 on children with CP; however, some evidence suggests that the disease has a disproportionately negative impact on this population. This impact includes the direct effect of the disease itself, in addition to the impact of the pandemic on the provided health services. A greater risk of developing a serious illness from COVID-19 is frequently observed in children with CP [33]. Furthermore, children with CP are challenged by barriers to access their usual healthcare, as well as other characteristics that increase their risk of COVID-19, such as reduced mobility, the need for direct care, and difficulty using preventive measures and communicating the symptoms of the illness [34].

The purpose of this study was to evaluate parental COVID-19 VH among parents of CP children in Egypt. The results of this study shed light on the prevalence of VH among parents of children with CP. Family income, parents’ previous COVID-19 infection, parents’ COVID-19 vaccination status, deaths among relatives due to COVID-19, willingness to vaccinate children against COVID-19, and receiving routine childhood vaccination were significant predictors of parents’ attitudes towards COVID-19 vaccination.

### 4.1. Vaccine Hesitancy

Approximately three-quarters of parents (70.6%) were hesitant to vaccinate their children with CP against COVID-19. Moreover, the vaccination rates against influenza in this population were consistently low (43.3%). In fact, many children with DDs are at a higher risk for complications from these respiratory pathogens [16]. Similarly, nearly 81.5% of Egyptian parents of children with chronic liver illnesses did not intend to vaccinate their children against COVID-19 [35]. Our finding is in line with studies conducted in Saudi Arabia that rated VH between 56% and 72% [36,37,38]. Similarly, Ali et al. [39] reported that 42.9% of Bengalis’ parents were hesitant about COVID-19 vaccination. In contrast, lower rates of VH were reported in Greece (8.9%) [40] and metropolitan areas in the United States (28%) [41]. It is worth noting that most of these studies recruited healthy children; this may explain the high VH rates among parents in the current study. Furthermore, previous studies suggest that high VH among adults in the East Mediterranean Region may explain our finding [42,43,44,45]. In addition, the safety and efficacy of the COVID-19 vaccine in children aged <12 years remain unclear, which might cause higher VH among parents of young children [46]. This may make parents provide self-treatment to their children in an attempt to prevent or treat COVID-19. It is worth noting that about 59.6% of the Egyptian population used self-treatment, without healthcare provider prescription, to treat COVID-19. They used either antibiotics, multivitamins, or herbal remedies. In fact, this risky behavior should be addressed and modified with vaccination to avoid antibiotic resistance and vitamin toxicity [47]. The successful implementation of vaccinations will depend on the cooperation between child pediatricians, disability-specific clinics, and occupational, physical, or speech experts who are familiar with the special needs of children with CP in their community. Therefore, in an attempt to improve vaccination in countries with high rates of VH, families must understand the advantages of the COVID-19 vaccine as an effective pandemic response and to protect these vulnerable children. Moreover, the vaccination of this high-risk group is crucial to resume receiving the required healthcare and returning to their schools.

### 4.2. Determinants of Parental Hesitancy Towards COVID-19 Vaccine

After adjusting for covariates, the results of the multiple linear regression analysis showed that parents with a COVID-19 infection had significantly higher PACV scores. Moreover, in this study, parents who perceived COVID-19 as a severe illness had a significantly positive attitude towards COVID-19 vaccination. However, this finding was not statistically significant in the regression analysis. It is reasonable to assume that parents’ favorable vaccination decisions are influenced by their perception of disease threats. In contrast, a previous study showed that Saudi parents’ refusal to vaccinate their children was significantly associated with their perception of COVID-19 as a rare disease, the belief that vaccination was not necessary, having a family member infected with COVID-19, had severe symptoms of COVID-19, and experiencing a COVID-19-related-death in the family [36].

Parents’ COVID-19 vaccination status was the most important predictor of VH. The PACV mean score was reduced six times if the parents received the COVID-19 vaccine. Similarly, a previous study found a positive association between parental willingness to get a vaccination and their reported intention to vaccinate their children [48,49]. Ennaceur et al. [37] found that parents who accepted vaccination had greater odds of vaccinating their children by 60%. Interestingly, Bell et al. [50] stated that parents were more likely to accept COVID-19 vaccinations for themselves than for their children.

The current study showed that high parental compliance with the childhood routine vaccines was negatively associated with VH. Additionally, parental willingness to vaccinate their children against COVID-19 was inversely related to the PACV scores. A similar finding was reported by Temsah et al. [51], Zhang et al. [52], and Goldmanet al. [53]. This is an expected preventive response by caring caregivers. Finally, non-Egyptian parents and those who could pay back their loans showed decreased likelihoods of VH. It could reflect the socioeconomic status of the parents, given the fact that economic hardship is a significant predictor of VH [54].

### 4.3. Limitations and Strengths of the Study

To the best of our knowledge, this is the first study to reveal the magnitude of VH and its associated factors among parents of children with CP. We conducted face-to-face interviews after administering a simple random sampling method to reduce selection bias, minimize non-responses, maximize data quality, and improve the generalizability of our findings. However, some limitations that may affect the generalizability of the results should be acknowledged. First, its cross-sectional design made it impossible to develop a causal relationship between the studied variables. Second, the lack of measurement of social and traditional media influence in this study may have confounded the results. Third, because the snapshot of vaccination intention was used in September 2022, attitudes and intentions toward vaccination may have changed over time. Therefore, a larger, multicenter longitudinal survey is needed.

## 5. Conclusions

Despite the fact that children with CP are at a higher risk of COVID-19, owing to the increased prevalence of underlying health conditions, we found that VH was prevalent among parents of children with CP. Herd immunity can be attained by implementing a variety of interventions to persuade parents to vaccinate their children. Incorporating the COVID-19 vaccine into the school vaccination program, disseminating clear and transparent information regarding the COVID-19 vaccine development process and the vaccine’s anticipated side effects, and establishing health promotion programs based on supportive parental attitudes and perceived behavioral control are all examples of interventions that can be implemented.

## Figures and Tables

**Table 1:** Characteristics of the parents (*n* = 321).

Variables		*n* (%)
Age (years)	18–29	58 (18.07)
	30–39	99 (30.84)
	40–49	101 (31.46)
	50–59	4 (19.63)
Relation to the Child	Mother	197 (61.37)
	Father	124 (38.63)
Nationality	Egyptian	280 (87.23)
	Non-Egyptian	41 (12.77)
Place of residence	Urban/City	271 (84.42)
	Rural	34 (10.59)
	Desert Region/mountains	16 (4.98)
Number of children	1	147 (45.79)
	2	128 (38.88)
	≥3	46 (14.33)
Education	High school or below	16 (4.98)
	University Degree	235 (73.21)
	Post graduate degree	70 (21.82)
Occupation	Government	150 (46.73)
	Private	141 (43.93)
	Not Employed	30 (9.35)
Employment	Work from home	24 (7.84)
	Part-time	79 (24.61)
	Full-Time	188 (58.57)
	Not Employed	30 (9.35)
Work sector	Health	57 (17.76)
	Non-Health	264 (82.24)
Health insurance	No	132 (41.12)
	Yes	189 (58.88)
Monthly income	Not enough, on a loan and cannot pay back	8 (2.49)
	Not enough, on a loan but can pay back	39 (12.15)
	Enough	237 (73.83)
	Enough and save	37 (11.53)
Older adults living in the same home	No	229 (71.34)
	Yes	92 (28.66)
Size of family	≤4 members	183 (57.1)
	5 members	72 (22.43)
	>5 members	66 (20.56)
Chronic disease	No	253 (78.82)
	Yes	68 (21.18)

**Table 2:** Characteristics of the children (*n* = 321).

Variables		*n* (%)
Age [Mean ± Standard Deviation (SD)]		8.2 ± 2.11
Gender	Male	142 (44.24)
	Female	179 (55.76)
Childbirth order	First	129 (40.31)
	Second	126 (39.38)
	Third	61 (19.06)
	≥fourth	4 (1.25)
Diagnosis	Hemiplegia	65 (20.25)
	Diplegia	132 (41.12)
	Quadriplegia	86 (26.79)
	Dyskinesia	19 (5.92)
	Ataxia	19 (5.92)
Gross Motor Function Classification System (GMFCS level)	I	62 (19.31)
	II	74 (23.05)
	III	62 (19.31)
	IV	43 (13.4)
	V	80 (24.9)
Previous COVID-19 infection	No	157 (48.91)
	Yes	45 (14.02)
	Maybe/not sure	119 (37.07)
Chronic disease	No	198 (61.68)
	Yes	123 (38.32)
Routine vaccines uptake	No	24 (7.48)
	Yes	297 (95.52)
Influenza vaccine uptake	No	182 (56.70)
	Yes	139 (43.40)

**Table 3:** Parental beliefs and attitudes towards COVID-19 and vaccination (*n* = 321).

Variables		*n* (%)
Parent’s previous COVID-19 infection	No	141 (43.9)
	Yes	158 (49.2)
	Not sure	22 (6.85)
COVID-19-related death in the family	No	190 (59.19)
	Yes	131 (40.81)
Parental COVID-19 vaccine status	Does not want to take the COVID-19 vaccine or continue the booster doses	208 (64.8)
	Already took the full doses or wants to continue to take the booster doses	113 (35.2)
The parents perceived COVID-19 as a dangerous illness (perceived seriousness)	Strongly Disagree	292 (90.97)
	Disagree	8 (2.49)
	Agree	20 (6.23)
	Strongly Agree	1 (0.31)
The parents suspected that their child would be at risk of COVID-19 (perceived susceptibility)	Moderate risk	121 (37.69)
	High risk	170 (52.96)
	Very high risk	30 (9.35)
Parental intention to vaccinate their children with the COVID-19 vaccine	No	205 (63.86)
	Yes	116 (36.14)

**Table 4:** Parental hesitancy towards COVID-19 vaccination using PACV (*n* = 321).

Covariates	PACV	*p*
	(Mean ± SD)	
Health Insurance		
No	37.06 ± 3.70	0.077
Yes	36.29 ± 3.86	
Income		
Not enough, on loan and cannot pay back	38.37 ± 2.87	
Not enough, on loan but can pay back	36.15 ± 3.96	0.076
Enough	36.81 ± 3.84	
Enough and save	35.37 ± 3.33	
Nationality		
Egyptian	36.93 ± 3.76	**<0.01**
Non-Egyptian	34.41 ± 3.38	
Parent’s previous COVID-19 infection		
No	36.39 ± 4.26	**<0.01**
Yes	36.45 ± 3.51	
Not sure	39.09 ± 1.02	
COVID-19-related death in the family		
No	36.05 ± 0.276	**<0.01**
Yes	37.38 ± 0.322	
Parental COVID-19 vaccine status		
Does not want to take the COVID-19 vaccine or continue the booster doses	38.69 ± 3.78	**<0.001**
Already took the full doses or wants to continue to take the booster doses	35.47 ± 3.32	
The parents perceived COVID-19 as a dangerous illness (perceived seriousness)		
Strongly Disagree	36.83 ± 3.84	**<0.01**
Disagree	35.5 ± 1.41	
Agree	33.6 ± 2.39	
Strongly Agree	41.0 ± 0	
Routine childhood vaccines uptake		
No	38.16 ± 2.69	**<0.01**
Yes	36.48 ± 3.86	
Parental intention to give COVID-19 vaccine to the child		
No	37. 95 ± 3.39	**<0.001**
Yes	34.24 ± 3.32	

Bold values denote statistical significance at the *p* < 0.05 level.

**Table 5:** Multilinear regression between the PACV score and different covariates (*n* = 321).

Covariates	β Coeff	*p*	95% CI
Health insurance			
No	Reference	0.08	−1.16, −0.07
Yes	−0.54		
Income			
Not enough, on loan and cannot pay back	Reference		
Not enough, on loan but can pay back	−2.39	**0.03**	−4.60, −1.89
Enough	−1.87	0.07	−3.91, 0.16
Enough and save	−1.09	0.33	−3.33, 1.13
Nationality			
Egyptian	Reference		
Non-Egyptian	−1.54	**0.002**	−2.53, −0.56
Parent’s previous COVID-19 infection			
No	Reference		
Yes	2.77	**<0.001**	1.94, 3.60
Not sure	2.94	**<0.001**	1.57, 4.32
COVID-19-related death in the family			
No	Reference		
Yes	−1.75	**<0.001**	−2.64, −0.87
Parental COVID-19 vaccine status			
Does not want to take the COVID-19 vaccine or continue the scheduled doses	Reference		
Fully vaccinated or wants to continue to take the scheduled doses	−6.28	**<0.001**	−7.41, −5.15
The parents perceived COVID-19 as a dangerous illness (perceived seriousness)			
Strongly Disagree	Reference		
Disagree	1.08	0.39	−1.44, 3.60
Agree	−1.04	0.11	−2.34, 0.25
Parental intention to give COVID-19 vaccine to the child			
No	Reference		
Yes	−3.04	**<0.001**	−3.75, −2.34
Routine Childhood vaccine uptake			
No	Reference		
Yes	−2.86	**<0.001**	−4.42, −1.30

Bold values denote statistical significance at the *p* < 0.05 level.

## Data Availability

The data collection tool used in this study is available as a Supplementary Material.

## Supplementary Materials

The following supporting information can be downloaded at: https://www.mdpi.com/article/10.3390/ijerph20031909/s1, The data collection tool is available in a supplementary file.

## Abbreviations

COVID-19	Coronavirus disease 2019
SARS-CoV-2	Severe acute respiratory syndrome coronavirus 2
DDs	Developmental disabilities
CP	Cerebral palsy
VH	Vaccine hesitancy
LMICs	Low-income and middle-income countries
WHO	World Health Organization
GMFCS	Gross Motor Function Classification System
OR	Odds ratio
CI	Confidence interval
