# Supplementary material for: Attitude of Parents of Children with Cerebral Palsy Towards COVID-19 Vaccination
Source: Int J Environ Res Public Health. 2023 Jan 20;20(3):1909. doi: 10.3390/ijerph20031909 (PMC9915268; doi:10.3390/ijerph20031909)
Supplement: Supplementary file 1 [file ijerph-20-01909-s001.zip › ijerph-2095506-supplementary.pdf]

Supplementary file

Attitude of parents of children with cerebral palsy towards the COVID-19 vaccine

You are invited to participate in research entitled" Attitude of parents of children with cerebral palsy towards the COVID-19 vaccine ". Your participation is voluntary and you are free to withdraw at any time. This study was approved by the Ethics Committee of the Faculty of Physical-Cairo University - Egypt, and there is no risk associated with participating in it. Confidentiality will be maintained.

1. **Serial**
2. **Diagnosis:**

Hemiplegia ☐ Diplegia ☐

Quadriplegia ☐ Dyskinesia ☐ Ataxia ☐

3. **GMFCS level:**

☐ I ☐ II ☐ III ☐ IV ☐ V

Sociodemographic data

4. **Nationality**
5. Place of residence
   a. Urban/ city
   b. Rural
   c. Desert region/ mountains
6. **Number of children between the ages of 5 and 12 years**
7. **Relation to the child**
   a. Mother
   b. Father
   c. Grandpa/Grandma

    d. Aunt or uncle
    e. Other:

8. **Can we state your age?**
    a. 18-29 years old
    b. 30-39 years old
    c. 40-49 years old
    d. 50-59 years
    e. More than 60 years
9. **Level of education**
    a. Reads and writes
    b. primary
    c. preparatory
    d. secondary
    e. University/college
    f. Postgraduate
10. **Place of work**
    a. Government
    b. Private
    c. Not Employed
11. **Type of employment**
    a. work from home
    b. (work part-time) 3 or 4 times a week every day
    c. Not employed
12. **Work Sector**
    a. Health
    b. Non-Health
13. **Health insured**
    a. Yes
    b. No
14. **Income/ month**
    a. Not enough, on a loan and cannot pay back
    b. Not enough, on a loan but can pay back
    c. Enough
    d. Enough and save

15. Older adults living in the same home

a. Yes
b. No

16. Family size

a. 2
b. 3
c. 4
d. 5
e. More than 5

17. Previous COVID-19 infection

a. Yes
b. No
c. Not sure

18. Previous death among relatives due to COVID-19

a. Yes
b. No

19. COVID-19 vaccine status

a. Do not want to take the vaccine
b. Took the first dose, awaiting for the second
c. Took the first dose but does not want to take the second dose
d. Took the first and second doses, awaiting for the booster dose
e. Took the first and second doses, but did not want to take the booster dose
f. Took the three doses
g. Want to take the vaccine, but it is not scheduled yet

20. Parent had chronic diseases

a. No
b. Blood pressure
c. Diabetes
d. Heart diseases
e. chest illness
f. Kidney and liver diseases

g. Rheumatic and immune diseases
h. Psychiatric and neurological diseases
i. Physical disability
j. Partial loss of hearing and vision

21. To what extent do you think that COVID-19 is a dangerous disease?

a. Strongly agree
b. Agree
c. Disagree
d. Strongly Disagree

Child's information

22. Age of the child (d/m/yrs)

23. Gender of the child

a. Male
b. Female

24.Child order

a. 1
b. 2
c. 3
d. 4
e. 5
f. 6

25. Children with previous COVID-19 Infection

a. Yes
b. No
c. I do not know

26. Children with chronic diseases

a. No
b. Blood pressure
c. Diabetes

d. Heart diseases
e. chest illness
f. Kidney and liver diseases
g. Rheumatic and immune diseases
h. Psychiatric and neurological diseases
i. Partial loss of hearing and vision

27. Did your child receive scheduled vaccines?

a. Yes
b. No
c. Not sure

28. Did your child receive influenza vaccine?

a. Yes
b. No
c. I do not know

29.If your child did not get the COVID-19 vaccine, do you have the intention to give him/ her the vaccine?

a. Yes
b. No
c. Maybe
d. Not applicable (In case your child got the vaccine)

30.To what extent do you think that your child can get COVID-19? *

	1	2	3	4	5	6	7	8	9	10	
غير متأكد علي الإطلاق	○	○	○	○	○	○	○	○	○	○	متأكد بشدة

Parent Attitudes About Childhood Vaccines (PACV)

31.Have you ever delayed having your child get COVID-19 vaccine for reasons other than illness or allergy?

a. Yes
b. No
c. Not sure

32. Have you ever decided not to have your child get COVID-19 vaccine for reasons other than illness or allergy?

a. Yes
b. No
c. Not sure

33.How sure are you that following the recommended shots of COVID-19 vaccine is a good idea for your child

a. Strongly agree
b. Agree
c. Disagree
d. Strongly Disagree

34.Children get more shots of COVID-19 vaccine that are good for them

a. Strongly agree
b. Agree
c. Not sure
d. Disagree
e. Strongly disagree

35.I believe that COVID-19 vaccine prevent many of severe illnesses

a. Strongly agree
b. Agree
c. Not sure
d. Disagree
e. Strongly disagree

36.It is better for my child to develop immunity by getting sick than to get a shot of COVID-19 vaccine

a. Strongly agree
b. Agree
c. Not sure
d. Disagree
e. Strongly disagree

37.It is better for children to get fewer shots of COVID-19 vaccine

a. Strongly agree

b. Agree
c. Not sure
d. Disagree
e. Strongly disagree

38. How concerned are you that your child might have a serious side effect from COVID-19 vaccine?

a. Not concerned at all
b. Not too concerned
c. Not sure
d. Somewhat concerned
e. Very concerned

39. How concerned are you that COVID-19 vaccine might not be safe for children?

a. Not concerned at all
b. Not too concerned
c. Not sure
d. Somewhat concerned
e. Very concerned

40. How concerned are you that COVID-19 vaccine might not prevent the disease?

f. Not concerned at all
g. Not too concerned
h. Not sure
i. Somewhat concerned
j. Very concerned

41. If you had another infant today, would you want him/her to get all the recommended shots of COVID-19 vaccine?

a. Yes
b. No
c. Not sure

42. Overall, how hesitant about childhood shots of COVID-19 vaccine would you consider yourself to be?

a. Not hesitant at all
b. Not too hesitant

c. Not sure
d. Somewhat hesitant
e. Very hesitant

43. I trust the information I receive about COVID-19 vaccine

a. Strongly agree
b. Agree
c. Not sure
d. Disagree
e. Strongly disagree

44. I am able to openly discuss my concerns about COVID-19 vaccine with my child's doctor

a. Strongly agree
b. Agree
c. Not sure
d. Disagree
e. Strongly disagree

45. All things considered; how much do you trust your child's doctor?

	1	2	3	4	5	6	7	8	9	10	
غير متأكد علي الإطلاق	◯	◯	◯	◯	◯	◯	◯	◯	◯	◯	متأكد بشدة

For any inquiry pls contact: Dr Ramy Ghazy
ramyghazy@yahoo.com
+201274741084
